# Do relaxed selection and habitat temperature facilitate biased mitogenomic introgression in a narrowly endemic fish?

**DOI:** 10.1002/ece3.2121

**Published:** 2016-04-29

**Authors:** Christopher Darrin Hulsey, Katherine L. Bell, Francisco J. García‐de‐León, Chris C. Nice, Axel Meyer

**Affiliations:** ^1^ Department of Biology University of Konstanz Universitätstraße 10 78457 Konstanz Germany; ^2^ Department of Biology Texas State University, San Marcos 601 University Drive 78666 San Marcos Texas; ^3^ Laboratorio de Genética para la Conservación Centro de Investigaciones Biológicas del Noroeste PO Box 128 La Paz B.C.S. Mexico

**Keywords:** Mexico, molecular convergence, sympatric speciation, trophic polymorphism

## Abstract

Introgression might be exceptionally common during the evolution of narrowly endemic species. For instance, in the springs of the small and isolated Cuatro Ciénegas Valley, the mitogenome of the cichlid fish *Herichthys cyanoguttatus* could be rapidly introgressing into populations of the trophically polymorphic *H. minckleyi*. We used a combination of genetic and environmental data to examine the factors associated with this mitochondrial introgression. A reduced representation library of over 6220 single nucleotide polymorphisms (SNPs) from the nuclear genome showed that mitochondrial introgression into *H. minckleyi* is biased relative to the amount of nuclear introgression. SNP assignment probabilities also indicated that cichlids with more hybrid ancestry are not more commonly female providing no support for asymmetric backcrossing or hybrid‐induced sex‐ratio distortion in generating the bias in mitochondrial introgression. Smaller effective population size in *H. minckleyi* inferred from the SNPs coupled with sequences of all 13 mitochondrial proteins suggests that relaxed selection on the mitogenome could be facilitating the introgression of “*H. cyanoguttatus*” haplotypes. Additionally, we showed that springs with colder temperatures had greater amounts of mitochondrial introgression from *H. cyanoguttatus*. Relaxed selection in *H. minckleyi* coupled with temperature‐related molecular adaptation could be facilitating mitogenomic introgression into *H. minckleyi*.

## Introduction

Hybridization can strongly influence animal diversification (Harrison 1986; Dowling and Secor 1997; Rüber et al. 2001; Seehausen 2004; Mallet 2005; Taylor et al. 2006; Parnell et al. 2012) and can be exceptionally important to the evolution of species with small ranges (López‐Pujol et al. 2012; Toews and Brelsford 2012). The intensity of selection, the frequency of genetic bottlenecks, and differences in population size can also lead to geographic asymmetries in introgression's role in diversification (Funk and Omland 2003; Riley et al. 2003; Chan and Levin 2005; Jones et al. 2012; Denton et al. 2014; Kovach et al. 2015). Introgression might even commonly lead to adaptation in species with small populations or restricted ranges because it could serve as a source of both novel genetic variation and subsequent phenotypic adaptation (Streelman et al. 2004; Meyer et al. 2006; Tobler and Carson 2010; Pardo‐Diaz et al. 2012; Kang et al. 2013). To determine what factors are associated with mitochondrial replacement in a classic case of trophic polymorphism, we used genetic and environmental data to examine introgression in the cichlid fish *Herichthys minckleyi*.

**Figure 1:**
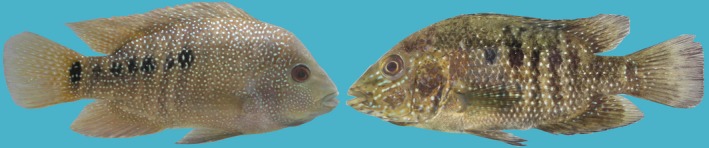
The cichlid species *Herichthys cyanoguttatus* (left) and *Herichthys minckleyi* (right) hybridize.

**Figure 2:**
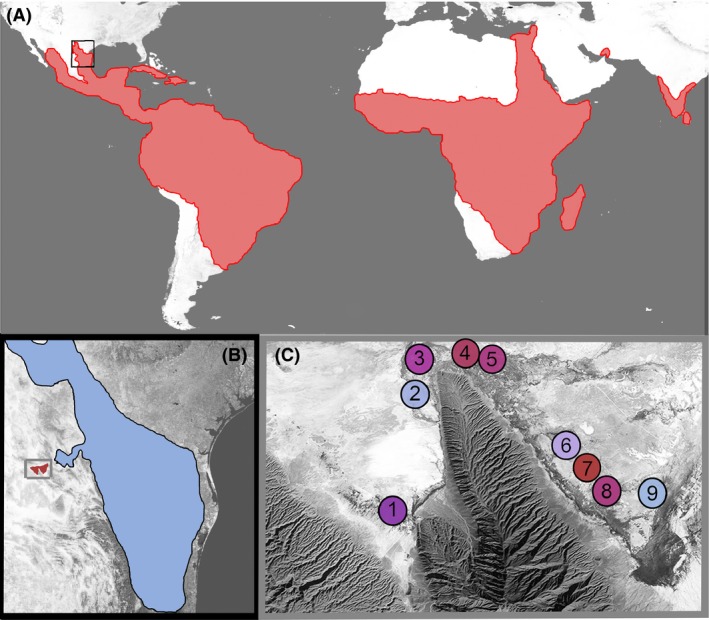
(A) The distribution of cichlids (red) abuts many temperate areas in sub‐Saharan Africa, southern India, the Levant, as well as the Neotropical regions of South and Central America. (B) *Herichthys cyanoguttatus* and *H. minckleyi* are the two cichlids in the Neotropics with the most northern ranges. In northeastern Mexico, the much larger range of *H. cyanoguttatus* (blue) meets the W‐shaped Cuatro Ciénegas Valley (dark red) where *H. minckleyi* is endemic. (C) There are approximately 200 springs in Cuatro Ciénegas containing cichlids, and the numbered circles circumscribe the locations where we sampled *H. minckleyi*. The springs are colored from light blue, colder, to dark red, hot, to mirror their temperatures (Table 1). These locations starting with the western lobe of the basin and moving around the Sierra de San Marcos mountain are given as: 1. Churince, 2. Juan Santos, 3. Tierra Blanca, 4. Mojarral Oeste, 5. Mojarral Este, 6. Escobedo, 7. Los Remojos, 8. Tío Candido, and 9. Los Gatos.

*Herichthys minckleyi* and its close relative *H. cyanoguttatus* (Fig. 1) inhabit a region of northeastern Mexico that provides an exceptionally tractable setting to test the patterns of divergence and gene flow (Fig. 2). The range of *H. cyanoguttatus* is huge, spanning 800 km from the Tropic of Cancer north to the Devils River in Texas. In contrast, *H. minckleyi* is restricted to the small (40 km by 40 km) Cuatro Ciénegas Valley in the Mexican Chihuahuan Desert (Minckley 1969). The entire distribution of *H. minckleyi* is composed of around 200 spring‐fed habitats that form a metapopulation of ecologically independent pools that are embedded within an inhospitable desert matrix (Chaves‐Campos et al. 2011a). Because *H. minckleyi* and *H. cyanoguttatus* have the most northern distribution of any cichlids in the Western Hemisphere (Minckley 1969; Miller et al. 2006), gene flow between *H. minckleyi* and a cichlid other than *H. cyanoguttatus* is also unlikely. Additionally, although these two species have been allopatric for approximately 2 million years (Hulsey et al. 2004), they can produce fertile hybrids in the laboratory (C.D. Hulsey unpubl.). Yet, despite being native to Río Grande tributaries 10 km outside Cuatro Ciénegas, *H. cyanoguttatus* likely only invaded the Cuatro Ciénegas Basin via man‐made canals in the last ~150 years (Hulsey and García de León 2013). This putatively recent gene flow following a long period of allopatric divergence suggests that the mechanisms structuring hybridization between the two species across their currently overlapping range could be readily disentangled.

*Herichthys minckleyi* is highly variable in its trophic morphology (Kornfield and Koehn 1975; Sage and Selander 1975; Kornfield and Taylor 1983; Trapani 2003; Hulsey et al. 2005; Hulsey 2006; Hulsey et al. 2008). This exceptional phenotypic variability coupled with the presence of highly differentiated (4% sequence divergence) *H. cyanoguttatus* mitochondrial haplotypes in ~30% of *H. minckleyi* suggests that hybridization could be playing a large role in *H. minckleyi* divergence (Hulsey and García de León 2013). However, the general extent of nuclear introgression remains unclear in this polymorphic species despite studies showing that mitochondrial introgression is common. Widespread mitochondrial introgression into *H. minckleyi* was first documented in Hulsey and García de León (2013). This study also found that two cichlids from Cuatro Ciénegas with “*H. cyanoguttatus”* mitochondrial haplotypes had stronger phylogenetic affinities at 84 nuclear loci with two individuals exhibiting the “*H. minckleyi*” mitochondrial haplotype than with a single *H. cyanoguttatus* individual from outside the valley. However, the limited sample size made generalizing the amount of mitochondrial introgression relative to nuclear introgression difficult. More recently, Magalhaes et al. (2015) also examined population subdivision of *H. minckleyi* with mitochondrial markers, but additionally examined 10 microsatellite loci from a number of locations within Cuatro Ciénegas. This study reaffirmed the widespread presence of “*H. cyanoguttatus*” mitochondrial haplotypes within *H. minckleyi,* but failed to delineate which, if any, microsatellite alleles were attributable to *H. cyanoguttatus* introgression. It currently remains unclear whether mitogenomic introgression from *H. cyanoguttatus* into *H. minckleyi* is substantially greater than the amount of nuclear introgression.

Discordance in the extent that mitochondrial and nuclear genes introgress is relatively common and has been documented in animal taxa as disparate as the *Drosophila yakuba* species complex (Bachtrog et al. 2006), Arctic and Brook charr (Doiron et al. 2002), North American darters (Bossu and Near 2009), and rainforest lizards (Singhal and Moritz 2012). This frequently observed bias in mitochondrial introgression could be due to a number of factors (Harrison 1989; Arnold 1993; Arnold and Meyer 2006). A breakdown in sexual recognition systems could result in asymmetric backcrossing of female *H. cyanoguttatus* with male *H. minckleyi* (Chan and Levin 2005). Also, although the sex‐determining mechanism in these cichlids is unknown, Haldane's rule would also predict that hybridization between *H. minckleyi* and *H. cyanoguttatus* could cause severe sex‐ratio distortion and biased production of females (Haldane 1922; Orr 1993). If males were the heterogametic sex, an abundance of female hybrids could be produced and result in the disproportionate maternal inheritance of *H. cyanoguttatus* mitochondria (Avise 1994; Ser et al. 2010; Jackel et al. 2013). Additionally, there could be selection on the metabolically critical mitochondrial locus due to abiotic factors such as temperature that vary extensively within the many isolated springs of Cuatro Ciénegas (Minckley 1969; Doiron et al. 2002; Funk and Omland 2003; Tobler and Carson 2010; Flight et al. 2011; Parmakelis et al. 2013). Although there is a danger of performing multiple comparisons when attempting to use the associations to isolate the mechanisms responsible for hybridization, high‐throughput methods of sampling the nuclear genome should allow us to extensively test which factors have led to any biases in *H. cyanoguttatus* mitochondrial introgression within the replicate aquatic environments of Cuatro Ciénegas.

In the much smaller range of *H. minckleyi* as compared to the wide‐ranging *H. cyanoguttatus*, the increased relevance of genetic drift could have also shifted the importance of selection (Darwin 1859; Lynch 2007; Ghalambor et al. 2015). Understanding why *H. cyanoguttatus* alleles are replacing those of *H. minckleyi* might necessitate considering the effective population sizes of the species and whether particular alleles have experienced relaxed selection regimes (Mallet 2005; Paland and Lynch 2006). Accounting for among‐site heterogeneity even during the molecular evolution of a single locus could also be critical for our understanding of why particular alleles like the *H. cyanoguttatus* mitochondria might be effective invaders (Pond et al. 2006; Wertheim et al. 2015). Because the mitochondrial genome contains numerous genes that are physically linked and do not recombine (Lynch 2007; Burton et al. 2013), a reduction in the intensity of selection on even one mitochondrial protein during *H. minckleyi*'s geographically unique history could have readily facilitated the invasion of the *H. cyanoguttatus* mitogenome.

Adaptation to temperature is not a broadly studied mechanism of cichlid evolution (but see Bootsma and Hecky 2003; Kavembe et al. 2014) although it is critical to the molecular evolution of the mitogenome (Silva et al. 2014; Welch et al. 2014). This lack of focus on temperature in cichlids has likely been due in part to the fact that these fish reach their peak diversity near the equator. However, their distribution does conspicuously end near the boundary between the tropics and the temperate zones on multiple continents (Fig. 2). Temperature could therefore extensively structure evolutionary processes at the temperate edges of the distribution of cichlids where species like *H. cyanoguttatus* are native. Interestingly, in a previous study of mitochondrial introgression into *H. minckleyi,* it was apparent that introgression was common in one of the coldest aquatic habitats in the valley, a pool named Juan Santos, and was nearly absent in one of the warmest habitats in the valley, the spring Escobedo (Evans 2005; Johnson et al. 2007; Hulsey and García de León 2013). Because *H. cyanoguttatus* is one of the more cold‐tolerant cichlids known and it occupies a region that experiences temperatures that would kill many other cichlid species (Hubbs 1951), its mitogenomic divergence from *H. minckleyi* could be substantially linked to temperature. Advantageously, a number of studies have already identified particular amino acids in mitochondrial proteins that are candidate adaptations to colder temperatures (Silva et al. 2014; Welch et al. 2014). If similar sites have diverged between *H. minckleyi* and *H cyanoguttatus*, this would bolster the idea that particular amino acid changes in the mitogenome are related to temperature. Also, because the springs that *H. minckleyi* inhabits are physically separated and largely isothermic throughout the year (Minckley 1969; Carson et al. 2012), these environments could be used as geographically dispersed replicates to test the importance of temperature during introgression into *H. minckleyi*.

We used a combination of genetic and environmental data to examine the factors associated with mitochondrial introgression into *H. minckleyi*. First, we used a data set of 6220 SNPs from the nuclear genome to ask whether the prevalence of the “*H. cyanoguttatus*” mitogenome within *H. minckleyi* is the result of biased introgression or whether rampant nuclear introgression is also occurring. We also used these SNPs to determine whether hybrid individuals are more likely to be female in order to test whether the patterns of nuclear introgression are consistent with mechanisms like asymmetric backcrossing or sex‐ratio distortion as a cause of biased mitogenomic introgression. Because the SNP data suggested that *H. cyanoguttatus* generally had a larger effective population size than *H. minckleyi* populations, we used sequences of all the protein‐coding genes in the mitochondria to determine whether relaxed selection could be a factor facilitating the introgression of mitochondrial haplotypes into *H. minckleyi*. Finally, we examined whether divergence in the mitogenomes of *H. minckleyi* and *H. cyanoguttatus* is characterized by similar amino acid changes as other vertebrate systems where temperature is mediating mitogenomic divergence and whether colder spring habitats were associated with greater mitochondrial introgression.

## Materials and Methods

### Phenotyping sex

We collected fish from the field as reported previously (Hulsey et al. 2006, 2015; Hulsey and García de León 2013). Following the collection, fin clips were stored in 100% ETOH for DNA analyses. Subsequently, the remaining whole fish specimens were preserved in formalin and then transferred to ethanol in the laboratory. We determined the sex of these *H. minckleyi* specimens (*n *=* *153) through dissection of the gonads under a light microscope as described in Hulsey et al. (2015). In brief, the gonads were removed from the body wall of the fish, and designations of sex were then based on previous studies of gonads in the closely related cichlid *Amphilophus citrinellus* (Oldfield 2011). Females were identified based on the presence of oocytes. Males were determined based on the lack of oocytes as well as the confirmation of the presence of spermatogenic cysts that are diagnostic for male reproductive structures. We then used a chi‐squared test to determine whether all female *H. minckleyi* sampled were more likely to exhibit the “*H. cyanoguttatus*” mitochondrial haplotype. Additionally, because spring habitats clearly showed structure in the amount of mitochondrial introgression, for the two springs where we recovered approximately 50% of individuals having each of the alternative mitochondrial haplotypes (Juan Santos and Tío Candido), we performed chi‐squared tests on the relationship between sex and mitochondrial haplotype.

### NGS amplified restriction fragment library

To assess the amount of nuclear introgression from *H. cyanoguttatus* into *H. minckleyi*, we generated a reduced representation library of the nuclear genome using a subset of the wild‐caught individuals from the Juan Santos (*n *=* *33) and Escobedo springs (*n *=* *35). The complete library consisted of 10 *H. cyanoguttatus* from outside the Cuatro Ciénegas Valley, 13 female *H. minckleyi* with “*H. cyanoguttatus*” haplotypes, 12 male *H. minckleyi* with “*H. cyanoguttatus*” haplotypes, 14 female *H. minckleyi* with “*H. minckleyi”* haplotypes, and 23 male *H. minckleyi* with “*H. minckleyi*” haplotypes. Mitochondrial data were unavailable for two individuals from Juan Santos and four individual from Escobedo, and therefore, the comparisons between the SNPs and mitochondria haplotype were reduced accordingly. To generate the SNP genotypes, we used a protocol that amplifies a reduced representation restriction fragment library from across the nuclear genome (Gompert et al. 2012; Parchman et al. 2012). For the library, extracted DNA was digested with EcoRI and MSE1 (New England Biolabs). Then, unique 10‐bp barcodes and Illumina adapter sequences were ligated to the fragments using T4 DNA ligase (New England Biolabs, Bio‐Rad, Qiagen: Ipswich, MA). These fragments were subsequently amplified via polymerase chain reaction (PCR) using iProof High‐Fidelity DNA polymerase (Bio‐Rad). The amplified fragments were pooled across individuals and separated on a 2% agarose gel. The fragments ranging from 200 to 500 bp were selected and purified using a QIAquick gel extraction kit (Qiagen). The resulting products were subsequently sequenced on one lane of an Illumina GAIIx sequencer (National Center for Genomic Resources, Santa Fe, NM).

The 125‐bp sequence reads generated were processed using custom Perl scripts that removed individual identifier sequences and restriction sites. Processed reads (109 bp) were assigned to individuals, assembled, and analyzed following the methods outlined in Parchman et al. (2012) and Gompert et al. (2012, 2014). Briefly, we performed a de novo assembly on the 15,800,517 sequence reads with SEQMAN NGEN (DNASTAR version 11.0.0.172, DNASTAR Inc.: Madison, WI) with a minimum match percentage of 92% and a minimum match size of 71 bp. Consensus reads from this assembly were pruned by removing excessively long (>113 bp), or short (<105 bp), sequences. Consensus reads were assembled using SEQMAN with a minimum match percentage of 83%, and any assembled sequences lower than this threshold were removed as potential paralogs. The resulting 142,618 consensus sequences were treated as scaffolds for reference‐based assembly. We mapped all the processed reads to the reference using the aln and samse algorithms in BWA version 0.7.5 (Li and Durbin 2009). We then created contigs using a maximum difference of two base pairs between the reference and sequence being aligned as well as a maximum gap of one. For all analyses, we only utilized reads that had a single best match. Variable sites were then called using SAMtools and BCFtools (Li et al. 2009). We required a minimum of 75% of individuals to have reads at a site in order for it to be called as a SNP. Any variable sites that included more than two alleles were excluded to avoid potential paralogs. We kept one randomly chosen variable site per contig and sorted variable sites into common (allele frequency > 5%) and rare variants (allele frequency ≤ 5%) using allele frequency point estimates obtained from BCFtools. To minimize erroneous SNP calls due to sequencing error, and to limit the rare SNPs that might be isolated to a few individuals or one locality, we focused on common variants and genotype likelihoods for these common loci. Rather than calling discrete genotypes, genotype likelihoods were retained and used in downstream analyses that integrate over genotype uncertainty (Gompert and Buerkle 2011; Gompert et al. 2012, 2014).

Single nucleotide polymorphism‐based assignment probabilities were estimated using the program ENTROPY, developed by Gompert et al. (2014). ENTROPY implements a Bayesian hierarchical model similar to that used in STRUCTURE (Pritchard et al. 2000; Falush et al. 2003). However, an important difference between ENTROPY and STRUCTURE is that ENTROPY incorporates sequence coverage, sequence error, and alignment error into a model that integrates over genotype uncertainty (Gompert et al. 2014). As with STRUCTURE, ENTROPY requires specification of the number of ancestral clusters (*k*) and does not incorporate prior information about which cluster an individual is assigned. Because we were specifically interested in the assignment probabilities to the two nominal species, *H. cyanoguttatus* and *H. minckleyi*, we used the model for *k* = 2. The probability of assignment to each species was then obtained using Markov chain Monte Carlo (MCMC). For each model, we ran five chains, 100,000 steps, a burn‐in of 10,000, and retained every 10th value. This resulted in 9000 samples from the posterior distribution for each chain (45,000 samples in total). MCMC chains were checked for convergence using the stabilization of trace plots and Gelman and Rubin's (1992) convergence diagnostic.

Because of the structure inherent among populations in Cuatro Ciénegas, we first used a *t*‐test to determine whether individuals from the two springs Juan Santos and Escobedo differed generally in their hybrid assignment probabilities in the same way they differed in their percentage of “*H. cyanoguttatus*” mitochondrial haplotypes. With a *t*‐test, we also examined the association between the presence of either a “*H. minckleyi*” or “*H. cyanoguttatus*” mitochondrial haplotype and assignment probabilities to the *H. cyanoguttatus* cluster in Juan Santos to determine whether mitochondrial ancestry predicted the amount of *H. cyanoguttatus* nuclear admixture within a population. This was not examined in Escobedo because we did not SNP genotype any individuals with the “*H. cyanoguttatus*” haplotype from this spring. Using a *t*‐test, we also determined the association between sexual phenotype and individual assignment probability in both springs. Importantly, because we were primarily interested in how admixture, inferred from the assignment probabilities, was associated with mitochondrial haplotypes or sex, modeling whether incomplete lineage sorting or hybridization was the explicit mechanism that produced the limited admixture observed was not essential to the hypotheses we tested. Finally, we used SAMtools and BCFtools (Li et al. 2009) to estimate genetic diversity for each population. We used the expectation–maximization algorithm, with 20 iterations, to estimate *π*, the expected heterozygosity, and Watterson's Θ, the effective population size, for *H. minckleyi* populations in both Juan Santos and Escobedo as well as for *H. cyanoguttatus* (Li 2011).

### Mitochondrial gene sequencing

We also generated mitochondrial protein‐coding sequences to explore the evidence for relaxed selection on *H. minckleyi*'s mitogenome. We utilized a combination of novel and previously published primers (Hulsey et al. 2013) to amplify all 13 protein‐coding genes of the mitogenome. To generate novel primers, we first created alignments of fully sequenced mitochondrial genomes of *Paraneetroplus synspilus* (NC_023526) and *Petenia splendida* (NC_024835) using the program Sequencher version 4.1 (Gene Codes, Ann Arbor, MI). We focused our primer design efforts on regions we identified by eye that ranged from 22 to 27 base pairs (bp) in length and that showed little divergence between these two cichlid species. We initially produced a series of forward and reverse primer pairs that spanned aligned regions from 1000 to 2000 bp in length. If PCR of these initial primer pairs failed to produce useful sequences, alternative primer pairs that spanned the regions but represented less conserved regions were generated. All primers were then realigned to the genomes to ensure that they did not have multiple priming sites.

For sequencing mitochondrial alleles, a 1.0 *μ*L aliquot of extraction was used to provide a DNA template for PCR. The master mix for all PCRs consisted of combining 13.0 *μ*L of GoTaq Hot Start Green Master Mix (Promega, Madison, WI), 2.5 *μ*L of both the forward and reverse primers, and 6.0 *μ*L of nuclease‐free water (Promega) for a final combined master mix volume of 24.0 *μ*L. Amplifications of the DNA template and master mix were carried out in a Perkin Elmer DNA thermocycler: Waltham, MA using standard methods. Thermal cycling conditions for all regions sequenced consisted of an initial denaturation step of 94°C (30 sec), an annealing step of either 55 or 49°C (30 sec), and an extension step of 72°C (1.5 min). A final elongation step of 72°C for 5 min was performed to end the PCR in order to ensure a complete extension of amplified products. Subsequently, the PCR products were electrophoretically separated from unincorporated primers and dNTPs using electrophoresis in low melting point agarose gel with ethidium bromide (1 mg/L) added and run in Tris–acetate buffer (pH 7.8). Positively amplified DNA was then enzymatically purified. The PCR products were sequenced using Sanger sequencing. Gene sequences were assembled from individual sequencing reactions using Sequencher version 4.1: Genecodes, Ann Arbor, MI.

For all 13 mitochondrial protein‐coding genes, we sequenced four *H. minckleyi* individuals that corresponded to the four major “*H. minckleyi*” haplotype groups identified previously based on CYTB (Table 2; Hulsey and García de León 2013). We also sequenced the protein‐coding loci in one *H. cyanoguttatus* from the Río Salado that drains into the Río Grande to contrast with the divergence in the “*H. minckleyi*” mitogenome. Additionally, we sequenced one cichlid from Cuatro Ciénegas that was recovered as having the “*H. cyanoguttatus”* allele in order to determine the amount of sequence divergence between the mitogenome of *H. cyanoguttatus* outside the valley and the mitogenome introgressed into *H. minckleyi*. Sequences are available on GenBank (Table S1). Finally, we surveyed the literature available on mitochondrial divergence in association with the temperature in vertebrates. We noted amino acid sites and residues that were inferred to be associated with the adaptation to temperature in the literature and highlighted putatively convergent amino acid replacements that differed between the mitochondrial proteins in the “*H. cyanoguttatus”* and “*H. minckleyi”* haplotypes.

### Mitochondrial relaxed selection analyses

**Figure 3:**
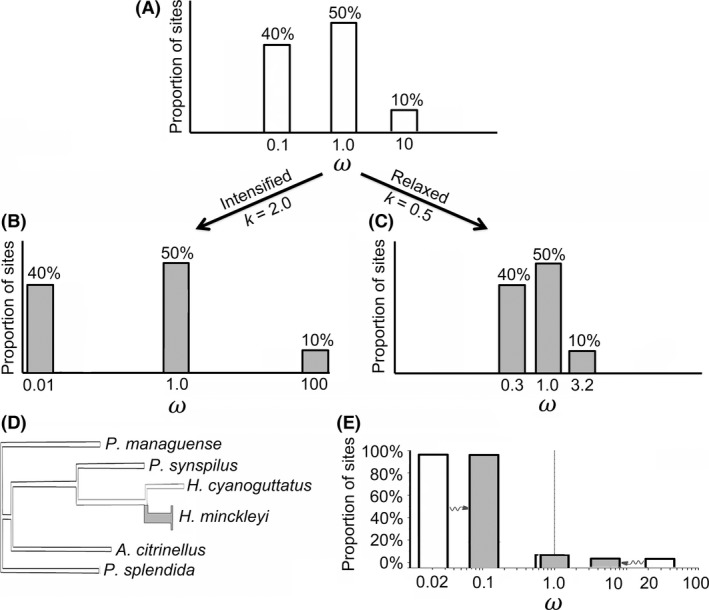
The reduction in the intensity of both purifying and positive selection might favor the invasion of novel alleles. To determine whether there was evidence of relaxed selection in the *H. minckleyi* mitogenome, we used the program RELAX (Wertheim et al. 2015). This model (A) groups amino acids into selection categories based on the patterns of synonymous and nonsynonymous substitution, *ω*. Some amino acids are assigned a *ω* that is >1.0 where there are more amino acid changes or nonsynonymous base pair changes than expected. Alternatively, other amino acids are assumed to be under neutral evolution *ω *= 1.0. Finally, others are categorized as having a *ω* that is much lower than one, indicative of purifying selection. Once all amino acids are assigned, it compares background patterns (white) of DNA substitution to a focal species of interest (gray). In the context of branch‐site models with multiple *ω* categories represented by different proportions of sites, selection can either increase (B) in intensity (*K* > 1.0) or decrease (C) in intensity (*K* < 1.0) in the focal species. When selection increases in intensity, *ω* values inferred for the selection categories can move away from neutral evolution (1.0). Alternatively, when selection is relaxed in intensity, sites under purifying selection increase toward a *ω* of 1.0, whereas sites under positive selection decrease toward 1.0. To examine the relaxation of selection along the branch leading to *H. minckleyi* (D), we reconstructed its mitochondrial protein evolution in the context of divergence of other Central American cichlids represented by *H. cyanoguttatus*,* Petenia splendida*,* Parachromis managuense*,* Amphilophus citrinellus,* and *Paraneetroplus synspilus*. (E) Most of the amino acid sites in the cichlid mitogenomes were inferred to be under purifying selection, and selection on the proteins of the mitochondria was significantly relaxed in *H. minckleyi*.

To test for evidence of relaxed selection in the mitogenome, we used the program RELAX (Wertheim et al. 2015). This model first groups amino acids into selection categories based on the patterns of synonymous and nonsynonymous substitution during the evolutionary history of closely related reference species (Fig. 3). Some amino acids have more amino acid changes, or nonsynonymous base pair changes, than expected. Other amino acids are assumed to be under neutral evolution having roughly equal synonymous and nonsynonymous changes. Finally, others are categorized as having less amino acid changes than expected, a result that is indicative of purifying selection. Once all amino acids are assigned, it compares the background patterns of DNA substitution to a focal branch leading to the species of interest and estimates the difference in the intensity of selection (*K*) between these branches. In the context of branch‐site models with multiple categories represented by different proportions of sites, selection can either increase in intensity (*K* > 1.0) or decrease in intensity (*K* < 1.0). When selection increases in intensity, values inferred for the selection categories tend to move away from neutral evolution. Alternatively, when selection is relaxed in intensity, sites under purifying and positive selection converge toward neutral evolution.

To generate an evolutionary context for the patterns of selection operating on the mitogenome in *H. minckleyi*, all new gene sequences generated were aligned with the protein‐coding regions of available mitogenomic sequences of other closely related Central American Heroine cichlids (Hulsey et al. 2004; Del Río‐Portilla et al. 2014). The reference species included were *P. splendida* (NC_024835), *Parachromis managuense* (NC_026918), *A. citrinellus* (NC_023827), and *P. synspilus* (NC_023526). Then, all base pairs corresponding to stop codons were trimmed from the end of the alignments of each gene. Using the Datamonkey online server that implements RELAX (Wertheim et al. 2015), a distance tree was constructed and the branch leading to all the *H. minckleyi* sequences from the last common ancestor of *H. cyanoguttatus* and all *H. minckleyi* sequences was designated as the focal branch. We initially analyzed the entire data set of 13 protein‐coding genes of the mitochondrial genome simultaneously to test for relaxed selection across the entire mitogenome of *H. minckleyi*. Then, we analyzed each individual protein to determine which particular genes, if any, exhibited evidence of relaxed selection.

### Temperature and mitochondrial introgression

We also determined how much of the variation in population levels (*n *=* *9) of mitochondrial introgression in Cuatro Ciénegas could be explained by spring water temperature (Table 1; Evans 2005; Johnson et al. 2007). To generate the level of mitochondrial introgression in a population, we combined new mitochondrial haplotype sequences (*n *=* *82) with published data for CYTB (*n *=* *180) from Hulsey and García de León (2013). This provided information on 262 haplotypes with an average of 29 individuals from each of the nine springs. Because the variability in introgression is often spatially structured, we first used a Mantel test to analyze whether geographic similarity could explain the amount of introgression. To account for potential spatial influences on introgression as well as temperature of springs, we then implemented a partial Mantel test to determine whether, once geography was accounted for, temperature could explain residual variation in population levels of mitochondrial introgression.

**Table 1:** The spring names and their western latitude (Lat [W]), northern longitude (Long [N]), temperature (°C), the number of mtDNA haplotypes sampled (*n*), and the percentage of “*H. cyanoguttatus*” haplotypes in each population

Spring	Lat (W)	Long (N)	°C	*n*	% Hybrid
Churince	102.08.20	26.50.53	31.1^1^	19^3^	11
Juan Santos	102.08.96	26.53.97	28.1^1^	40^3^	80
Tierra Blanca	102.08.43	26.55.39	32.9^2^	39^4^	0
Mojarral Oeste	102.07.50	26.55.47	34.2^2^	72^3^	15
Mojarral Este	102.07.32	26.55.46	33.5^2^	18^3^	28
Los Remojos	102.06.67	26.55.01	29.7^1^	11^3^^,^ 4	45
Escobedo	102.05.20	26.53.48	34.7^1^	29^3^	3
Tío Candido	102.04.85	26.52.33	32.0^1^	26^3^^,^ 4	31
Los Gatos	102.09.17	26.51.17	25.5^1^	8^4^	100

Two sources were used for the temperature of the springs (^1^Evans 2005; ^2^Johnson et al. 2007). The mtDNA haplotypes were taken from two studies (^3^Hulsey and García de León 2013; ^4^this study).

## Results

**Figure 4:**
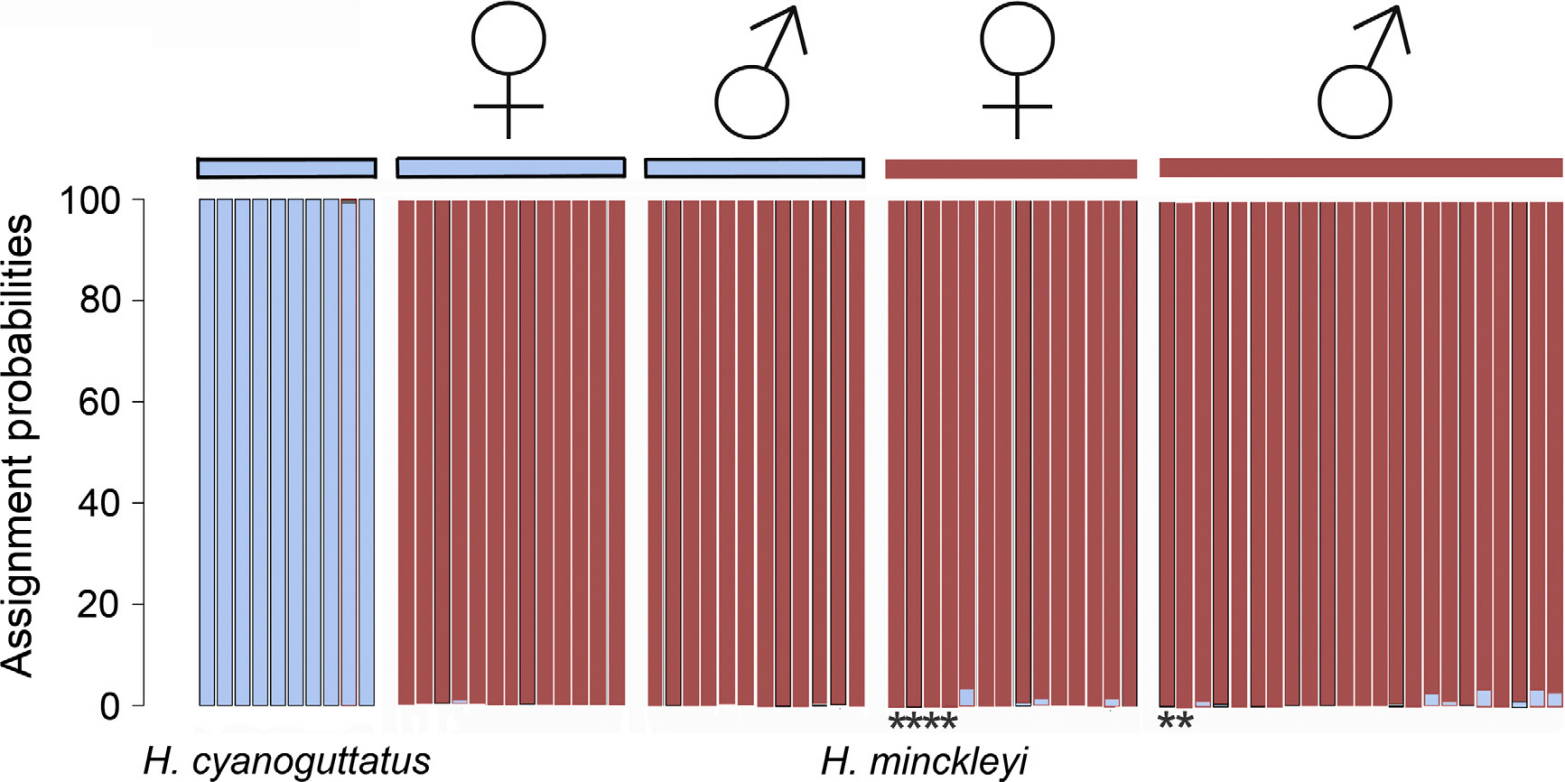
Clustering of *H. cyanoguttatus* and *H. minckleyi* based on 6220 SNP markers. Despite the widespread sharing of mtDNA haplotypes, the nuclear genome clearly genetically delineates *H. cyanoguttatus* individuals (blue cluster) from all *H. minckleyi* individuals (red cluster). There was no support for females exhibiting greater hybrid ancestry. All individuals with the “*H. cyanoguttatus*” mitochondrial haplotype (blue rectangle above the assignment probabilities) examined in the SNP analyses were taken in Juan Santos. Individuals with the “*H. minckleyi*” mitochondrial haplotype (red rectangle above the assignment probabilities) were mostly from the spring Escobedo, except for six individuals from Juan Santos (*).

The reduced representation library produced 6220 common SNPs. The common SNP‐based assignment probabilities of *H. minckleyi* to *H. cyanoguttatus* ranged from 0.0 to 3.2%, suggesting that there was little overall nuclear introgression of *H. cyanoguttatus* into *H. minckleyi* (Fig. 4). However, the two springs did differ in the amount of inferred nuclear introgression (*P *<* *0.001), with Juan Santos cichlids showing significantly less (0.038 ± 0.001%) probability of assignment to *H. cyanoguttatus* than cichlids in Escobedo (0.759 ± 0.011%). This contrasts strongly with Juan Santos having a much higher proportion of introgressed “*H. cyanoguttatus*” mitochondrial haplotypes than Escobedo (Table 1). Within Juan Santos, there was no association between mitochondrial haplotype and an individual's SNP assignments to *H. cyanoguttatus*. There was also no support for females being more likely to be hybrid based on the “*H. cyanoguttatus*” mitochondrial haplotype (*χ* ^2^ **=* *1.217; *P *=* *0.270) when all 153 individuals were analyzed together. When analyzed in the two springs with close to equal abundance of “*H. cyanoguttatus*” and “*H. minckleyi*” haplotypes, females were also not more likely to have the “*H. cyanoguttatus*” haplotype in either Tío Candido (*χ* ^2^ **=* *2.968; *P *=* *0.085) or Juan Santos (*χ* ^2^ **=* *0.734 *P *=* *0.391). Female *H. minckleyi* also did not have a higher proportion of their nuclear genome being assigned to *H. cyanoguttatus* as compared to male *H. minckleyi* in either spring (Juan Santos: *P *=* *0.757; Escobedo: *P *=* *0.846). Based on the SNPs, *H. cyanoguttatus* had lower heterozygosity (*π *= 0.0028), but a larger effective population size (*Θ *=* *0.0037), than either population of *H. minckleyi* (Juan Santos: *π *= 0.00349; *Θ* = 0.00210 and Escobedo: *π *= 0.00348; *Θ* = 0.00207).

The sequence divergence between mitochondrial proteins in *H. minckleyi* and *H. cyanoguttatus* was substantial (Table 2). The 13 proteins were on average 4.0% sequence divergent between all four “*H. minckleyi”* and the “*H. cyanoguttatus*” mitogenomes. Sequence divergence between the two species ranged from 5.4% for ND3 to 2.8% in COX2. There were a very large number of, 71, amino acid changes (1.9% of all amino acid sites sequenced) that consistently distinguished the “*H. cyanoguttatus*” haplotype from all four “*H. minckleyi*” haplotypes (Table 3). In line with *H. cyanoguttatus* having recently invading Cuatro Ciénegas, the two “*H. cyanoguttatus*” sequences were only 0.19% divergent (Table 2). There were also no amino acid differences in mitochondrial proteins recovered between the *H. cyanoguttatus* sampled outside the valley and the “*H. cyanoguttatus*” mitogenome sequenced from the *H. minckleyi* in the center of the Cuatro Ciénegas Valley (Table 3). However, there were seven amino acid replacements that were present in only one or more of the *H. minckleyi* that were not shared with the other *H. minckleyi* and *H. cyanoguttatus*. The genes ATP8 and ND4L did not have any amino acid differences among any of the “*H. minckleyi*” and “*H. cyanoguttatus*” sampled. With 19, ND5 had the most amino acid replacements between *H. cyanoguttatus* and *H. minckleyi,* and this gene also exhibited four of the intraspecific *H. minckleyi* mitogenomic amino acid differences. The amino acid differences between *H. minckleyi* and *H. cyanoguttatus* at site 155 in COX3, 368 in CYTB, and 12 in ND4 are locations that have been implicated in mitogenomic cold adaptation in other vertebrates (Silva et al. 2014; Welch et al. 2014).

**Table 2:** The uncorrected combined mitochondrial protein sequence divergence between “*H. minckleyi*” and “*H. cyanoguttatus*” haplotypes. Ordered from east to west in the Cuatro Ciénegas Valley, the “*H. minckleyi*” samples correspond to samples taken from the springs Tío Candido (IV), Escobedo (I), Los Remojos (II), and Churince (III). The Roman numerals correspond to major CYTB haplotypes identified in Hulsey and García de León (2013). The sample from Mojarral East is a “*H. cyanoguttatus*” haplotype present in a *H. minckleyi* individual from the middle of the Cuatro Ciénegas Valley. The Río Salado sample is from the native range of *H. cyanoguttatus*

Springs	Tío Candido	Escobedo	Churince	Los Remojos	Mojarral East	Río Salado
Tío Candido						
Escobedo	0.05%					
Churince	0.19%	0.23%				
Los Remojos	0.22%	0.26%	0.13%			
Mojarral East	3.77%	3.77%	3.70%	3.70%		
Río Salado	4.00%	4.00%	3.90%	3.90%	0.19%	

**Table 3:** Divergence between *H. minckleyi* and *H. cyanoguttatus* in the 13 protein‐coding genes of the mitochondrial genome is described in their linked order in the cichlid mitogenome

Gene	% Div	*K*	*P*	AA sites	AA Δ
ND1	5.0	0.89	0.600	293 (5)	27(G⇔A), 51(V⇔I), 87(A⇔T), 115(I⇔V), 204(T⇔A)
ND2	4.3	0.71	0.181	348 (10)	6(L⇔S), 95(T⇔A), 148(A⇔T), 206(M⇔T), 238(A⇔T), 239(L⇔I), 241(T⇔A), 243(A⇔T), 331(M⇔L), 332(A⇔A)
COX1	3.1	0.62	0.082	520 (2)	477(A⇔T), 492(A⇔V)
COX2	2.8	0.42	0.019	230 (5)	27(A⇔T), 123(V⇔I), 129(T⇔A), 183(A⇔T), 191(I⇔V)
ATP8	3.2	22.70	0.590	53 (0)	None
ATP6	5.6	0.99	0.979	227 (9)	14(Y⇔H), 21(T⇔A), 29(I⇔V), 65(G⇔G), 71(A⇔V), 136(V⇔T), 189(V⇔I), 194(V⇔A), 195(T⇔I)
COX3	2.6	0.64	0.915	261 (1)	155(H⇔R)^1^
ND3	5.4	0.21	0.342	116 (5)	13(I⇔V), 20(T⇔A), 31(D⇔N), 80(D⇔N), 94(V⇔A)
ND4L	3.5	13.91	0.360	98 (0)	None
ND4	4.1	0.40	0.014	460 (10)	12(V⇔I)^2^, 15(A⇔T), 17(A⇔T), 29(A⇔T), 47(D⇔N), 48(T⇔M), 186(M⇔T), 198(A⇔T), 213(A⇔V), 406(A⇔T)
ND5	4.1	0.57	0.042	612 (23)	8(L⇔M), 31(L⇔P), 32(P⇔L), 39(N⇔D), 51(V⇔T), 64(T⇔A), 72(N⇔N), 93(T⇔A), 97(I⇔V), 118(Y⇔H), 206(I⇔V), 209(T⇔A), 210(S⇔P), 227(T⇔A), 278(T⇔T), 355(S⇔S), 433(I⇔V), 479(T⇔T), 498(A⇔T), 502(A⇔T), 520(V⇔A), 554(N⇔S), 573(I⇔V)
ND6	3.2	1.02	0.981	173 (2)	121(T⇔S), 136(V⇔V)
CYTB	4.1	0.68	0.024	378 (6)	209(A⇔V), 233(A⇔V), 329(V⇔I), 333(V⇔I), 348(I⇔T), 368(T⇔A)^3^
All mtDNA proteins	4.0	0.71	0.003	3769 (78)	

The percent sequence divergence (%Div) between the “*H. cyanoguttatus”* haplotype collected from Mojarral Este in the center of the Cuatro Ciénegas Valley and the “*H. minckleyi”* haplotype sampled from *H. minckleyi* in Escobedo is presented here as exemplars. The relaxation parameter (*K*) and the significance (*P*) of relaxed selection along the branch leading to *H. minckleyi* for mtDNA proteins as inferred from RELAX (Wertheim et al. 2015) are shown. The number of amino acid sites examined for each protein (AA sites) as well as the number of divergent amino acids between the “*H. minckleyi*” and “*H. cyanoguttatus*” proteins is given in parentheses. The values for all mtDNA proteins analyzed simultaneously are also shown. The position of amino acid differences (AA Δ) numbered from the start codon for each protein is given with standard abbreviations for amino acids that differ between *H. cyanoguttatus* (first amino acid) and *H. minckleyi* (second amino acid). Amino acid changes related to mitochondrial temperature adaptation in the COX3 and ND4 genes of polar bears (^1,2^Welch et al. 2014) and CYTB in anchovies (^3^Silva et al. 2014) are noted.

The protein‐coding component of the *H. minckleyi* mitogenome appears to have experienced relaxed selection (Fig. 3; Table 3). The combined selection analysis of all protein‐coding loci in *H. minckleyi* relative to the background evolution of other cichlid mitogenomes supports the hypothesis that the mitogenome has undergone a relaxation in the intensity of selection (*K* = 0.71; *P *=* *0.003). Most of the proteins had a *K* value <1.0, but statistically indistinguishable from 1.0. However, the two shortest genes in the mitogenome, ATP8 (*K* = 22.70; *P *=* *0.590) and ND4L (*K* = 13.91; *P* = 0.360), were inferred to show a tendency, although not significant at *P* < 0.05, toward intensifying selection. However, the genes ND5 (*K* = 0.57, *P* = 0.042), CYTB (*K* = 0.68; *P* = 0.024), COX2 (*K* = 0.42; *P* = 0.019), and ND4 (*K* = 0.40; *P* = 0.014) all exhibited a significant signature of relaxed selection.

Temperature in the springs ranged extensively from a high of 34.7°C in Escobedo to 25.5°C in Los Gatos (Table 1). The springs in Cuatro Ciénegas also differed substantially in the estimated amount of mitochondrial introgression (Table 1). From lowest to highest, the percent introgression for each spring was as follows: Tierra Blanca (0%), Escobedo (3%), Churince (11%), Mojarral Oeste (15%), Mojarral Este (28%), Tio Candido (31%), Los Remojos (45%), Juan Santos (80%), and Los Gatos (100%). The Mantel test suggested that there was weak evidence for isolation by distance determining haplotype frequencies of “*H. cyanoguttatus*” alleles in springs (*P *=* *0.058). Despite some of the variation in mitochondrial introgression being accounted for by geography, the partial Mantel test that used geography as a covariate indicated that temperature explains a significant component of the variation among springs in “*H. cyanoguttatus*” haplotype frequency (*P *=* *0.038).

## Discussion

Introgression of the mitochondrial genome of *H. cyanoguttatus* into *H. minckleyi* is far more extensive than introgression from the nuclear genome (Fig. 4; Hulsey and García de León 2013; Magalhaes et al. 2015). Despite many *H. minckleyi* having a “*H. cyanoguttatus*” mitochondrial haplotype, nuclear markers unambiguously delineate *H. minckleyi* and *H. cyanoguttatus*. No *H. cyanoguttatus* individuals were assigned to the cluster containing all *H. minckleyi* in Cuatro Ciénegas. Additionally, the probability of assigning the SNP‐inferred nuclear genome of a “*H. minckleyi*” mitochondrial individual to *H. cyanoguttatus* was <4% in all cichlids examined. The definitive nuclear genome assignments of both *H. minckleyi* and *H. cyanoguttatus* to discrete clusters make it clear that the *H. minckleyi* we examined are not simply taxonomically misidentified *H. cyanoguttatus*. Additionally, the negative association between the probability of being assigned to the *H. cyanoguttatus* population and the possession of a “*H. cyanoguttatus*” mitochondrial haplotype among the two spring populations examined suggests that different forces are likely structuring mitochondrial and any nuclear introgression in *H. minckleyi*.

The strictly maternal inheritance of mitochondria causes biases in its introgression (Arnold 1993). Hybridization can cause severe sex‐ratio distortion leading to an overabundance of females that results in a breeding pool more likely to pass on maternally inherited hybrid mitochondria (Ser et al. 2010; Jackel et al. 2013). Yet, the lack of any cichlids collected in Cuatro Ciénegas with even close to equal proportions of *H. minckleyi* and *H. cyanoguttatus* SNP assignment probabilities suggests that any cichlids we examined were unlikely to be F1 hybrids. If they are hybrids, they are more likely to be the product of multiple generations of backcrossing. Therefore, our analyses might provide a weak test of the importance of hybrid sex‐ratio distortion to mitochondrial introgression, as hybrid‐induced skewed sex ratios would be expected to last no more than a few generations following the initial hybridization (While et al. 2015). Breakdown in sexual recognition systems could also result in asymmetric backcrossing of female *H. cyanoguttatus* with male *H. minckleyi* (Chan and Levin 2005). However, we found no support for sex of Cuatro Ciénegas cichlids being associated with a greater degree of either mitochondrial introgression or nuclear introgression (Fig. 4). The bias in mitochondrial introgression observed in *H. minckleyi* might have little to do with intrinsic incompatibilities and more to do with genetic drift or extrinsic environmental factors.

The very limited (0.19%) genetic divergence between the introgressed “*H. cyanoguttatus*” mitogenome in Cuatro Ciénegas cichlids and *H. cyanoguttatus* outside the valley (Table 2) indicates that the mitochondrial invasion is likely very recent. This sequence divergence is less than the divergence among some of the “*H. minckleyi*” haplotypes within Cuatro Ciénegas. Like a number of other documented cases of hybridization (Daniels and Corbett 2003; Riley et al. 2003; Streelman et al. 2004), the introgression of *H. cyanoguttatus* mitogenomes into *H. minckleyi* could be human mediated. It seems feasible that the ~150‐year‐old canals that now connect this previously isolated valley with the Río Grande drainage could have provided a conduit for gene flow between *H. cyanoguttatus* and *H. minckleyi* (Hulsey and García de León 2013). Prior to this connection, Cuatro Ciénegas likely had no natural outflow for at least the last several hundred thousand years (Minckley 1969; Chaves‐Campos et al. 2011b). Also, *Herichthys* species tend to produce hundreds of offspring during a single breeding event, breed multiple times a year, and can become reproductively active within about 9 months (Kornfield et al. 1982; Miller et al. 2006). Therefore, during the last century, the man‐made alterations to the Cuatro Ciénegas Valley coupled with the reproductive life history of these cichlids could easily have facilitated an extensive and relatively rapid introgression of *H. cyanoguttatus* mitochondrial genotypes into *H. minckleyi*.

Nonadaptive scenarios could help to explain the biased mitochondrial introgression into *H. minckleyi*. Extensive introgression can follow from purely neutral processes that are primarily related to demographic shifts (Excoffier and Ray 2008). The lower heterozygosity but higher effective population size estimates of *H. cyanoguttatus* based on our SNP genotypes are consistent with a recent range expansion for this invading species (Excoffier et al. 2009; Jezkova et al. 2015). This type of range expansion could also lead to “surfing” of mitochondrial alleles as a wave of introgression into a species like *H. minckleyi* (Edmonds et al. 2004).

Relaxation of selection could also be playing a major role in the mitochondrial invasion into *H. minckleyi* (Mallet 2005; Higham et al. 2015; Wertheim et al. 2015). The small range of *H. minckleyi* and the spatial population structure of the springs of Cuatro Ciénegas (Fig. 2) both could increase the chances of individuals mating with close relatives and translate into a smaller effective population size (Lynch 2007). This is likely one cause of the relaxed selection on the mitogenome observed in *H. minckleyi* relative to other Central American cichlids (Table 3). The smaller effective population size could have translated into the enhanced accumulation of mutations in the *H. minckleyi* mitochondrial genome due to the more pronounced effects of genetic drift (Wertheim et al. 2015).

With over 70 amino acid replacements found between the *H. cyanoguttatus* and *H. minckleyi* mitochondrial proteins, these compact genomes also have accumulated ample divergence that could provide the substrate for adaptation to different selective regimes. Additionally, although there is a substantial variation in temperature among the springs in Cuatro Ciénegas (Table 1), each individual pool is likely relatively isothermic. If the mitogenome in these fishes is important for adaptation not just to a particular temperature but also to temperature fluctuations, the local constancy of water temperatures in the spring‐fed Cuatro Ciénegas habitats could have similarly resulted in relaxed selection on the *H. minckleyi* mitochondria. Because *H. cyanoguttatus* faces both extreme summer heat and freezing winter air temperatures in the streams and rivers that encompass much of its range, its native environment is exceptionally temperate for a cichlid (Hubbs 1951). In contrast, the lack of environmental variation and metabolic demands on regulating temperature via the mitogenome in *H. minckleyi* could have contributed to relaxed selection on its mitogenome.

Similarities in amino acid composition between other vertebrates that have diverged along a climatic gradient also suggest that the *H. cyanoguttatus* mitogenomic invasion into *H. minckleyi* could be related specifically to the lower temperature in some springs (Table 2). The amino acid differences between *H. minckleyi* and *H. cyanoguttatus* at site 155 in COX3 and 12 in ND4 are locations that have been implicated in mitochondrial cold adaptation in polar bears (Welch et al. 2014). Even more suggestive for the importance of temperature in mitogenomic divergence is amino acid site 368 in CYTB. This amino acid shows a strong association with the temperature adaptation in anchovies in the Atlantic Ocean (Silva et al. 2014). Furthermore, this amino acid site exhibits a threonine in *H. cyanoguttatus* and a valine in the colder regions of the anchovies range. But, there is an alanine residue both in the putatively warm‐adapted “*H. minckleyi”* haplotypes and in the warmer part of the anchovies range. However, because putative examples of convergence like these are dependent in part on what has been investigated in other studies of mitochondrial adaptation and the change of particular amino acids within the mitogenome is likely highly nonrandom, it is difficult to know whether these putative cases of convergence exceed our expectation of what might differ randomly between the two cichlid mitogenomes. Nevertheless, as the relation between temperature and molecular evolution is investigated in more detail in many other species, our understanding of these types of putatively convergent molecular adaptations will increase.

Finally, there is a correlation between the population‐level percentage of introgressed “*H. cyanoguttatus*” haplotypes and the temperature of springs in Cuatro Ciénegas. The environmental gradient among these springs is substantial with the warmer springs that *H. minckleyi* inhabits representing the higher end of temperatures that any aquatic vertebrate is known to exploit (Minckley 1969; Carson et al. 2012). Importantly, selection on the mitochondria of individuals might not simply translate into differential survival among the various temperature regimes. For instance, it is possible that the putatively more cold‐adapted *H. cyanoguttatus* is better able to breed in colder habitats leading to the observed preponderance of their mitochondria in these springs. These possibilities could be explored using field studies of survivorship and observations of breeding in different springs in Cuatro Ciénegas (Fitzpatrick and Shaffer 2004; Chunco 2014; Chown et al. 2015). Additionally, future studies could examine heat‐shock proteins or other genes involved in metabolism that reside in the nuclear genome to determine whether the introgression of these temperature‐related genes follows patterns similar to the introgression observed here for the mitogenome (Burton et al. 2013; Parmakelis et al. 2013). Patterns of introgression across metapopulations such as the spring systems of Cuatro Ciénegas could provide a powerful framework for understanding how spatial gradients influence hybridization and adaptation in this system.

Introgressive hybridization is increasingly thought to contribute to diversification in general, and it might be exceptionally important to highly diverse groups like cichlids (Rüber et al. 2001; Seehausen 2004; Mallet 2005; Pardo‐Diaz et al. 2012; Parnell et al. 2012; Kang et al. 2013). Yet, because most cichlid assemblages contain a large number of recently diverged and frequently sympatric species that could hybridize (Fryer and Iles 1972; Meyer 1990, 1993; Smith and Skulason 1996; Streelman et al. 2004; Kautt et al. 2012; Machado‐Schiffiano et al. 2015), determining both the source species and factors structuring the introgression of hybrid genotypes could be difficult (Mims et al. 2010). In contrast, the substantial independent evolutionary history, metapopulation structure, and a minimal number of congeners in locations such as Cuatro Ciénegas could provide ideal systems to allow us to determine what are the major factors structuring introgression.

## Data Accessibility

Mitochondrial DNA sequences have been deposited on the GenBank nucleotide database (Table S1). The reduced representation Illumina reads are available on the NCBI SRA database (SAMN04523166).

## Conflict of Interest

None declared.

## Supporting information


**Table S1.** The Genbank numbers for the cichlid mitochondrial protein DNA sequences.
