# Supplementary material for: Do relaxed selection and habitat temperature facilitate biased mitogenomic introgression in a narrowly endemic fish?
Source: Ecol Evol. 2016 Apr 29;6(11):3684–98. doi: 10.1002/ece3.2121 (PMC4853310; doi:10.1002/ece3.2121)
Supplement: Supplementary file 1 — Table S1. The Genbank numbers for the cichlid mitochondrial protein DNA sequences. [file ECE3-6-3684-s001.docx]

Supplementary Table 1. The Genbank numbers for the cichlid mitochondrial protein DNA sequences. KN precedes all numbers on Genbank. The genes are listed as column headings. The Mojarral East individual possessed the “*Herichthys cyanoguttatus*” mitochondrial genome but was a *H. minckleyi.* The Río Salado individual was an *H. cyanoguttatus* collected from the native range of *H. cyanoguttatus*. All other individuals correspond to *H. minckleyi* with “*H. minckleyi*” mitochondrial genomes.

|  | **ATP6** | **ATP8** | **COX1** | **COX2** | **COX3** | **CYTB** | **ND1** | **ND2** | **ND3** | **ND4** | **ND4L** | **ND5** | **ND6** |
| --- | --- | --- | --- | --- | --- | --- | --- | --- | --- | --- | --- | --- | --- |
| Mojarral East | 156838 | 354512 | 340685 | 210427 | 210435 | 222921 | 340692 | 272182 | 279584 | 272180 | 222928 | 222933 | 340675 |
| Churince | 156839 | 354514 | 340681 | 210428 | 210434 | 222922 | 340691 | 272187 | 279581 | 222922 | 222926 | 222934 | 340676 |
| Río  Salado | 156840 | 354511 | 340686 | 210429 | 210436 | 222920 | 340689 | 272183 | 279585 | 272176 | 222931 | 222935 | 340677 |
| Tío Candido | 156841 | 354508 | 340682 | 210430 | 210437 | 222924 | 340688 | 222936 | 279582 | 272177 | 222925 | 222936 | 340678 |
| Escobedo | 156842 | 354510 | 340683 | 210431 | 210438 | 340683 | 340687 | 272184 | 279583 | 272178 | 222929 | 222937 | 340679 |
| Los Remojos | 156843 | 354509 | 340684 | 210432 | 210439 | 222923 | 340690 | 272185 | 279580 | 272179 | 222930 | 222938 | 340680 |
